# A membrane‐associated NAC transcription factor OsNTL3 is involved in thermotolerance in rice

**DOI:** 10.1111/pbi.13297

**Published:** 2019-12-06

**Authors:** Xue‐Huan Liu, Yu‐Shu Lyu, Weiping Yang, Zheng‐Ting Yang, Sun‐Jie Lu, Jian‐Xiang Liu

**Affiliations:** ^1^ State Key Laboratory of Genetic Engineering School of Life Sciences Fudan University Shanghai China; ^2^ State Key Laboratory of Plant Physiology and Biochemistry College of Life Sciences Zhejiang University Hangzhou China; ^3^ National Key Laboratory of Crop Genetic Improvement Huazhong Agricultural University Wuhan China; ^4^ School of Life Sciences Guizhou Normal University Guiyang China

**Keywords:** membrane‐associated transcription factor, *Oryza sativa*, OsNTL3, OsbZIP74, thermotolerance

## Abstract

Heat stress induces misfolded protein accumulation in endoplasmic reticulum (ER), which initiates the unfolded protein response (UPR) in plants. Previous work has demonstrated the important role of a rice ER membrane‐associated transcription factor OsbZIP74 (also known as OsbZIP50) in UPR. However, how OsbZIP74 and other membrane‐associated transcription factors are involved in heat stress tolerance in rice is not reported. In the current study, we discovered that OsNTL3 is required for heat stress tolerance in rice. Os*NTL3* is constitutively expressed and up‐regulated by heat and ER stresses. Os*NTL3* encodes a NAC transcription factor with a predicted C‐terminal transmembrane domain. GFP‐OsNTL3 relocates from plasma membrane to nucleus in response to heat stress and ER stress inducers. Loss‐of‐function mutation of Os*NTL3* confers heat sensitivity while inducible expression of the truncated form of OsNTL3 without the transmembrane domain increases heat tolerance in rice seedlings. RNA‐Seq analysis revealed that OsNTL3 regulates the expression of genes involved in ER protein folding and other processes. Interestingly, OsNTL3 directly binds to *OsbZIP74* promoter and regulates its expression in response to heat stress. In turn, up‐regulation of Os*NTL3* by heat stress is dependent on OsbZIP74. Thus, our work reveals the important role of OsNTL3 in thermotolerance, and a regulatory circuit mediated by OsbZIP74 and OsNTL3 in communications among ER, plasma membrane and nucleus under heat stress conditions.

## Introduction

Global climate change, including an increase in average temperatures and atmospheric CO_2_ concentrations, has great impacts on plant growth and yield production (Lesk *et al.*, 2016; Lobell *et al.*, 2011). It has been predicted that the extreme annual daily maximum temperature will increase by about 1 to 3 ºC by the mid twenty‐first century (IPCC, 2012). As a result, a yield loss of 6 to 7% per 1  ºC increase in seasonal mean weather associated with extreme heat disasters was estimated (Lesk *et al.*, 2016). Being sessile, plants have evolved to cope with the fluctuating environmental temperature conditions with complex and interconnected signalling pathways essential for growth and development (Bita and Gerats, 2013; Bokszczanin *et al.*, 2013). Understanding of the molecular mechanisms underlying plant responses to heat stress is important for engineering more tolerant crops to high temperature.

Under heat stress conditions, the major injuries in plants are the loss of membrane integrity/fluidity and protein stability (Bita and Gerats, 2013). When misfolded proteins are accumulated in cytosol, the classical heat shock response (HSR) is activated to either increase protein folding efficiency or eliminate misfolded protein accumulation. During HSR in plants, disruption of the interaction between heat shock proteins (HSPs) and heat shock transcription factors (HSFs) at high temperature leads to sequential activation of HSFs and up‐regulation of downstream genes encoding HSPs and other factors for enhancing protein folding capacity (Nover *et al.*, 2001; Ohama *et al.*, 2016).

Heat stress also causes the accumulation of misfolded proteins in endoplasmic reticulum (ER), triggering the unfolded protein response (UPR) (Liu and Howell, 2010). In the model plant *Arabidopsis*, UPR is mediated by several ER membrane‐associated basic leucine zipper (bZIP) transcription factors, for example AtbZIP28 and AtbZIP60 (Liu and Howell, 2016). When misfolded proteins are accumulated in ER, the transmembrane domain of AtbZIP28 is cleaved via intramembrane proteolysis by the Golgi‐resident protease site‐2 protease (S2P), and the activated AtbZIP28 relocates to the nucleus to up‐regulate downstream genes (Che *et al.*, 2010; Gao *et al.*, 2008; Liu *et al.*, 2007; Liu *et al.*, 2007; Sun *et al.*, 2015; Tajima *et al.*, 2008; Zhou *et al.*, 2015). In contrast, activation of the ER membrane‐associated AtbZIP60 depends on unconventional mRNA splicing of At*bZIP60* controlled by the ER‐localized Inositol‐requiring enzyme 1 (AtIRE1), and an open reading frame shift resulted from splicing leads to nuclear localization of AtbZIP60 (Deng *et al.*, 2011; Moreno *et al.*, 2012; Nagashima *et al.*, 2011). Knock‐outs of AtbZIP28 and AtbZIP60 confer high sensitivities to heat stress at reproductive stages in terms of silique length and seed setting in *Arabidopsis* (Zhang *et al.*, 2017).

The rice (*Oryza Sativa *L*.*) ortholog of AtbZIP60, OsbZIP74 (also known as OsbZIP50), is also activated in response to ER stress. Under normal growth conditions, Os*bZIP74* mRNA encodes an ER membrane‐associated bZIP transcription factor. Under ER stress conditions, the conserved double stem‐loop structure in Os*bZIP74* mRNA encoding hydrophobic amino acids is spliced out, which is dependent on the ER‐localized OsIRE1 protein. Thereafter, the spliced Os*bZIP74* mRNA has a reading frame shift and encodes a nucleus‐localized OsbZIP74 (Hayashi *et al.*, 2012; Lu *et al.*, 2012). The unconventional splicing of Os*bZIP74* mRNA is also induced by heat stress and salicylic acid treatment in rice (Lu *et al.*, 2012). Two other rice membrane‐associated bZIP transcription factors, OsbZIP17 (also known as OsbZIP39) and OsbZIP16 (also known as OsbZIP60), are the homologs of AtbZIP17 and AtbZIP28, both of which regulate downstream gene expression during ER stress response in rice (Hayashi *et al.*, 2013; Takahashi *et al.*, 2012). However, whether these ER stress regulators are important for abiotic stress tolerance such as heat stress tolerance are not demonstrated.

In the current study, we found that OsbZIP74 up‐regulates the expression of *OsNTL3* in response to heat and ER stresses. Os*NTL3* encodes a plasma membrane‐associated NAC transcription factor that relocates to the nucleus and regulates the expression of Os*bZIP74* and other genes involved in UPR under heat stress conditions. Genetic analysis demonstrated that mutation of Os*NTL3* confers high heat sensitivity while inducible expression of the truncated form of OsNTL3 increases heat stress tolerance in rice seedlings. Thus, OsNTL3 is important for thermotolerance through relaying heat stress signals/effects from plasma membrane to nucleus.

## Results

### Loss‐of‐function of Os*NTL3* confers heat sensitivity in rice

**Figure 1:**
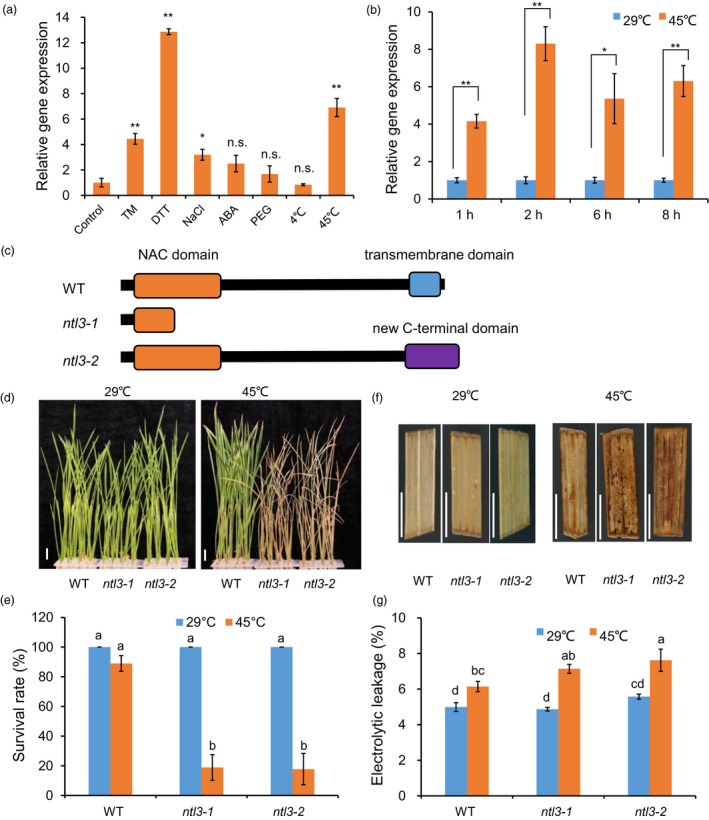
Loss‐of‐function of Os*NTL3* confers heat stress sensitivity in rice. (a–b) Up‐regulation of Os*NTL3* by ER stress and abiotic stresses. Eight‐day‐old wild‐type rice Nipponbare seedlings were treated with various stresses (TM, 5 μg/ml; DTT, 2 mm; NaCl, 250 mm; ABA, 0.1 mm; PEG 4000,15%) for 4 hr and roots were harvested for Os*NTL3* expression analysis (a). Time‐course experiments were performed under heat stress conditions (b). Relative gene expression is the expression level of Os*NTL3* in stressed plants relative to that in non‐stressed plants, both of which were normalized to that of the internal control *ACTIN*. Error bars represent SE (*n* = 3). Asterisks indicate significance levels when comparing to the control in t‐test. (*, *P* < 0.05; **, *P* < 0.01; n.s., not significant at *P* < 0.05). (c‐e) Heat stress phenotypes of the Os*NTL3* mutant plants. Eight‐day‐old wild‐type (WT) seedlings and two lines of targeted‐gene‐edited Os*NTL3* (*ntl3‐1* and *ntl3‐2*) mutant seedlings grown at 29 ºC were transferred to 45 ºC for 5 days and then photographed after recovering at 29 ºC for 7 days (d). Survival rate was count based on the appearance of newly developed green leaves (e). Totally, 141 rice plants under each temperature condition for each genotypes were examined for phenotype analysis. Protein domain structures of OsNTL3 in plants were depicted in c. (f–g) ROS accumulation and electrolytic leakage in WT and Os*NTL3* mutant plants under different temperature conditions. Four‐week‐old wild‐type (WT) seedlings and Os*NTL3* gene‐edited mutant seedlings grown at 29 ºC were transferred to 45 ºC for 16 hr, leaves were sampled for DAB staining (f) or transferred to 45 ºC for 5 hr, and leaves were sampled for electrolyte conductivity measurements (g). Error bars represent SE (*n* = 6 in e and *n* = 3 in g). Different letters indicate significant differences in comparisons between two samples as determined by LSD test following ANOVA analysis (*P* < 0.05). Bar = 1 cm.

Genome‐wide analysis showed that there are at least 13 and 6 membrane‐associated NAC transcription factors in Arabidopsis and rice, respectively, most of which are up‐regulated by abiotic stresses (Kim *et al.*, 2007). However, the functions of most of these proteins in rice are less understood. We were interested in characterization of the biological function of Os*NTL3* (LOC_Os01g15640) in rice. To examine the effects of abiotic stresses on Os*NTL3* expression, wild‐type rice seedlings were subjected to various stress treatments, including ER stress inducers tunicamycin (TM) and dithiothreitol (DTT), salt stress (NaCl), osmotic stress (PEG), phytohormone (ABA) and temperature stresses (4 ºC and 45 ºC), and the expression of Os*NTL3* was analysed by quantitative RT–PCR (qPCR). Beside the constitutive expression of Os*NTL3* in the assayed samples, the expression of Os*NTL3* was significantly (FC > 2, *P* < 0.05) up‐regulated by TM, DTT, salt and heat stress treatments (Figure 1a). Time‐course experiment further confirmed the heat stress effects on the expression of Os*NTL3* in rice (Figure 1b).

To explore the possible role of Os*NTL3* in heat stress response in rice, we generated several gene‐edited loss‐of‐function lines of Os*NTL3* with the CRISPR‐Cas9 system. Two of them, *ntl3‐1* and *ntl3‐2*, were characterized in details in the current study. Os*NTL3* is predicted to encode a premature truncated protein in the *ntl3‐1* mutant and a truncated version plus a new C‐terminus in the *ntl3‐2* mutant (Figure 1c and Figure S1), Both *ntl3‐1* and *ntl3‐2* mutant seedlings grew as normally as WT plants under standard (29 ºC) growth temperature conditions (Figure 1d‐e). However, when these plants were subjected to heat stress treatment (45 ºC for 5 days), compared with WT plants, both *ntl3‐1* and *ntl3‐2* mutant plants were more sensitive to heat stress treatment as reflected by the survival rates after recovery at 29 ºC (Figure 1d‐e). We concluded that Os*NTL3* is important for heat stress tolerance in rice seedlings.

### OsNTL3 relocates from plasma membrane to nucleus in response to heat stress

**Figure 2:**
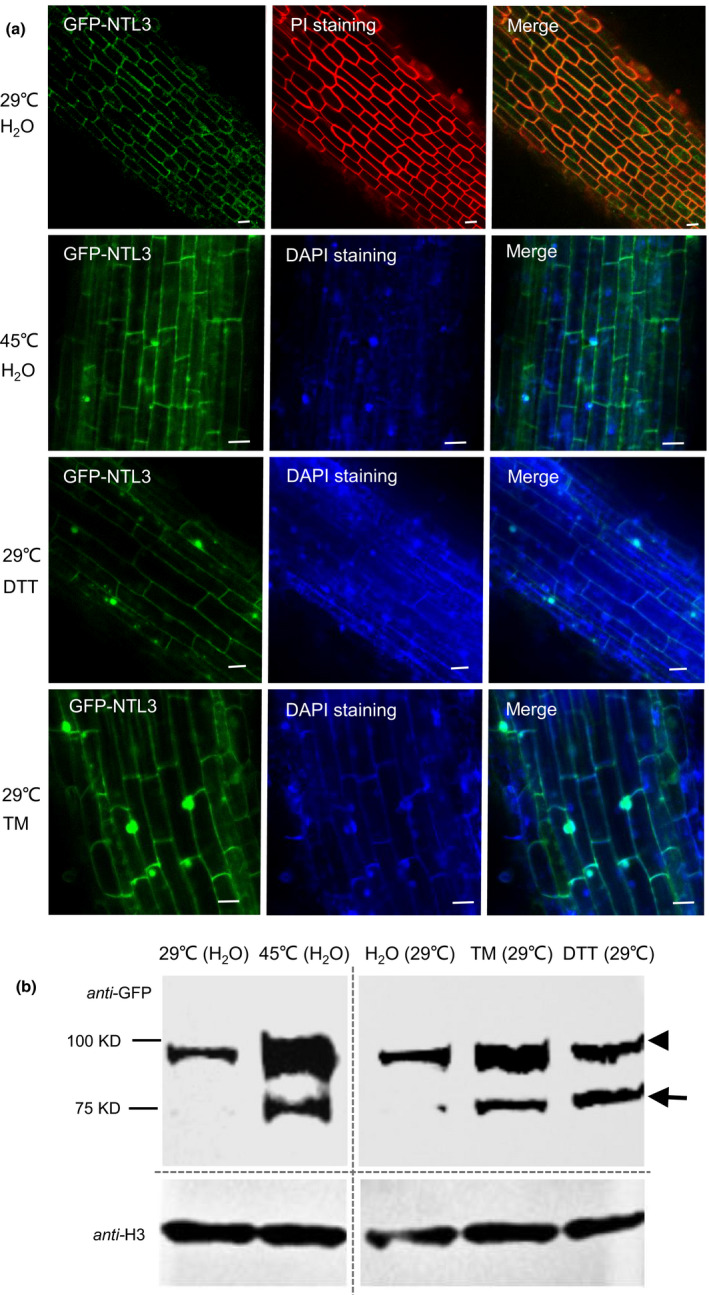
OsNTL3 is processed in response to heat and ER stresses in rice. (a) Nuclear relocation of GFP‐NTL3 in response to heat and ER stresses. PI and DAPI staining were used to show the cell boundary and nucleus, respectively. Bar = 50 μm. (b) Western blot analysis of the processing of GFP‐NTL3 in rice seedlings in response to heat and ER stresses. Transgenic rice plants overexpressing the full‐length GFP‐NTL3 were treated without (29 ºC) or with heat (45 ºC), DTT and TM for 2 hr, and root cells were checked under confocal microscopy (a) or Western blot analysis (b). Molecular weight markers are listed on the left and a‐H3 serves as the loading control. Arrow head and arrow point to the precursor and processed form, respectively.

To investigate the subcellular localization of OsNTL3, we fused GFP to the N‐terminus of full‐length OsNTL3 and expressed it in stably transformed rice plants via agrobacteria‐mediated transformation. Under normal temperature growth conditions, most of the GFP‐NTL3 signals were observed in the cell boundaries in root tips, as shown with the Propidium Iodide (PI) staining (Figure 2a). In contrast, after heat stress treatment, GFP‐NTL3 signals were frequently found in nuclei overlaid with 4',6‐diamidino‐2‐phenylindole (DAPI) staining (Figure 2a). Relocation of GFP‐NTL3 from plasma membrane to nucleus in response to ER stresses (TM and DTT treatment) was also observed in root cells in rice (Figure 2a). OsNTL3 contains a transmembrane domain at C‐terminus, which is predicted to be cleaved before its activation (Seo *et al.*, 2008). To check the possible processing of OSNTL3, we performed Western blotting analysis with the *GFP‐OsNTL3* overexpression rice plants. When non‐stressed rice plants were sampled, GFP‐NTL3 migrated as a major high molecular weight band (~ 95 kD); however, when rice plants were subjected to heat stress or ER stress treatments, beside the high molecular weight form, another low molecular weight band (~75 kD) was observed in the Western blotting analysis, and the abundance of precursor band was also increased (Figure 2b). These results suggested that OsNTL3 is partially processed and relocates from plasma membrane to the nucleus in response to heat and ER stresses.

### OsNTL3 regulates heat stress responsive genes in rice

**Figure 3:**
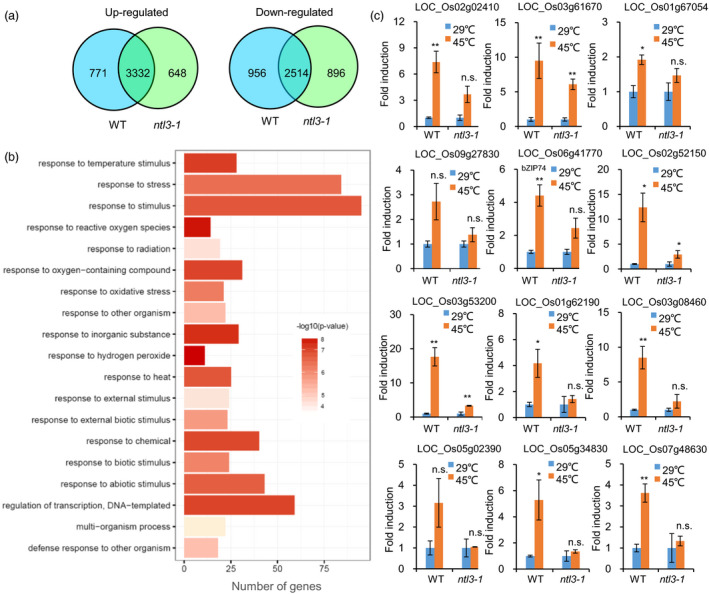
OsNTL3 regulates heat responsive genes in rice. (a) Venn diagrams showing the numbers of overlapping and non‐overlapping genes that were up‐regulated or down‐regulated in WT and *ntl3‐1* mutant plants, respectively. Eight‐day‐old rice seedlings were subjected to heat stress at 45 ºC for 3 hr, and gene expression profiles were checked by RNA‐Seq analysis. Criteria for differential expression were set as *q* ≤ 0.05, fold change (FC) ≥2 for up‐regulation or FC ≤ 0.5 for down‐regulation. (b) GO analysis of Os*NTL3*‐dependent heat up‐regulated genes. Totally, 463 Os*NTL3*‐dependent genes whose expression (FPKM) in heat‐stressed WT was more than 10 were used for GO analysis. (c) Validation of Os*NTL3*‐dependent gene expression under heat stress conditions in *ntl3‐1* mutant in rice. Eight‐day‐old WT and *ntl3‐1* rice mutant seedlings grown at 29 ºC were treated at 45 ºC for 5 h, and total RNA was extracted for qRT‐PCR. Fold induction is the expression level of Os*NTL3* in stressed plants relative to that in non‐stressed plants, both of which were normalized to that of the internal control *ACTIN*. Error bars represent SE (*n* = 3). Asterisks indicate significance levels when comparing to the control in *t*‐test. (*, *P* < 0.05; **, *P* < 0.01; n.s., not significant at *P* < 0.05).

OsNTL3 belongs to the plant‐specific NAC transcription factor family (Shao *et al.*, 2015). To examine whether OsNTL3 regulates gene expression under heat stress conditions, we firstly checked the transcriptional activation activity of OsNTL3 in yeast cells. Four truncated forms of OsNTL3 were fused to GAL4 DNA‐binding domain, respectively (Figure S2a). Compared with the empty vector control, all the fusion proteins except NTL3∆4 (AA 1‐137) had transcriptional activation activity in yeast cells (Figure S2b). These results suggested that OsNTL3 is a transcriptional activator, and its transcriptional activity depends on the region from D137 to N354. The mutated form of OsNTL3 obtained from the *ntl3‐2* mutant was also used in the study. It was found that this mutated form does not have transcriptional activation activity (Figure S2c). To find out Os*NTL3*‐regulated genes during heat stress response, we compared gene expression profiles in WT and *ntl3‐1* mutant plants with or without heat stress treatment using RNA‐Sequencing (RNA‐Seq) analysis. In the WT plants, totally 4103 and 3470 genes were significantly up‐regulated (log_2_FC ≥ 1, q ≤ 0.05) and down‐regulated (log_2_FC ≤ −1, q ≤ 0.05), respectively (Figure 3a and Data S1). Among them, 771 genes were not significantly up‐regulated (log_2_FC ≥ 1, q ≤ 0.05) and 956 genes were not significantly down‐regulated (log_2_FC ≤ −1, q ≤ 0.05) in the *ntl3‐1* mutant plants (Figure 3a and Data S1). Because OsNTL3 is a transcriptional activator, we considered these 771 up‐regulated genes as Os*NTL3*‐dependent heat stress responsive genes.

Further Gene Ontology (GO) enrichment analysis showed that above‐mentioned 771 genes are enriched in responses to hydrogen peroxide, reactive oxygen species and temperature stimulus, et al. (Figure 3b). We checked reactive oxygen species (ROS) accumulation in WT and Os*NTL3* mutant plants subjected to heat stress. We found that more H_2_O_2_ was accumulated in *ntl3‐1* and *ntl3‐2* mutant plants than that in WT plants under heat stress conditions (Figure 1f). Higher electrolytic leakage was also found in the Os*NTL3* mutant plants than that in WT plants in response to heat stress (Figure 1g).

We selected 12 genes from the gene expression data set and performed qRT‐PCR. These genes, encoding LOC_Os02g02410 (ER protein chaperone BiP1), LOC_Os03g61670 (calreticulin), LOC_Os01g67054 (calreticulin), LOC_Os09g27830 (protein disulfide isomerase), LOC_Os06g41770 (OsbZIP74), LOC_Os02g52150 (heat shock 22 kDa protein), LOC_Os03g53200 (calmodulin‐related calcium sensor protein), LOC_Os01g62190 (C2H2 zinc finger protein), LOC_Os03g08460 (AP2 domain‐containing protein), LOC_Os05g02390 (C2H2 zinc finger protein), LOC_Os05g34830 (NAC domain‐containing protein) and LOC_Os07g48630 (ethylene‐insensitive 3), were up‐regulated (fold change> 2, *P* < 0.05) by heat stress in WT rice seedlings (Figure 3c). However, the fold induction rates of these genes were reduced or not significant (*P* < 0.05) in *ntl3‐1* mutant plants (Figure 3c). Thus, Os*NTL3* regulates several heat responsive genes in rice, especially those genes involved in UPR.

### OsNTL3 directly regulates the expression of Os*bZIP74* in response to heat stress

**Figure 4:**
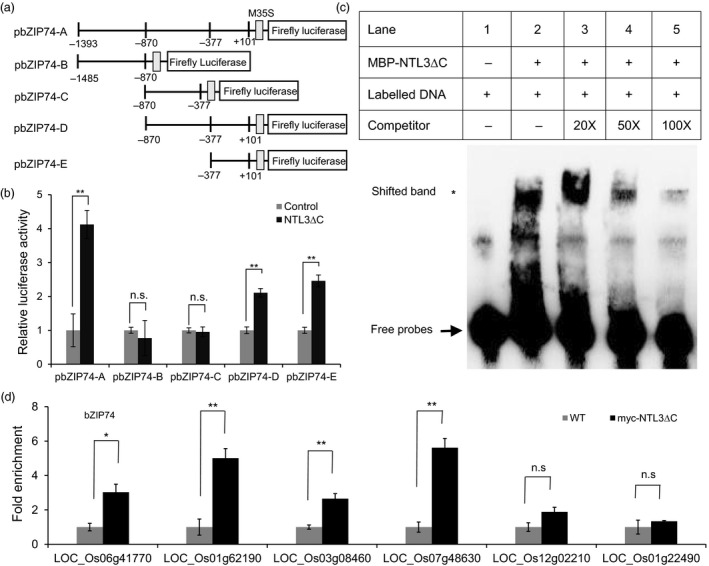
OsNTL3 directly controls the promoter activity of Os*bZIP74*. (a‐b) Activation of the Os*bZIP74* promoter by OsNTL3. Various DNA fragments of Os*bZIP74* promoter were linked to the minimal 35S promoter (M35S) and the reporter firefly luciferase (a). The position relative to the transcription start site is indicated below. (b) The processed forms of OsNTL3 (NTL3∆C) was co‐expressed with various reporters in tobacco leaves in the dual‐luciferase assays. The relative luciferase activity is the firefly luciferase activity normalized to the Renilla luciferase activity driven by the 35S constitutive promoter, which was then normalized to the empty vector control. (c) Direct binding of OsNTL3 to Os*bZIP74* promoter sequences. MBP‐tagged truncated form of OsNTL3 (MBP‐NTL3∆C) was incubated with the biotin‐labelled Os*bZIP74* promoter DNA (pbZIP74E). Different concentrations of non‐labelled cold probes were used as competitors to show the binding specificity. Star and arrow point to the positions of shifted bands and free probes, respectively. (d) Enrichment of myc‐NTL3∆C to the promoter regions of target genes. Fold enrichment is the immuno‐precipitation efficiency in the NTL3 ∆C‐myc transgenic rice plants normalized to that in the WT plants. LOC_Os12g02210 and LOC_Os01g22490 are two OsNTL3‐independent genes used as negative controls. Error bars represent SE (*n* = 3). Asterisks in c and d indicate significance levels when comparing to the control in t‐test. (*, *P* < 0.05; **, *P* < 0.01; n.s., not significant at *P* < 0.05).

Since up‐regulation of Os*bZIP74* is affected in *ntl3‐1* mutant plants (Figure 3c), we were interested to know whether OsNTL3 directly regulates the expression of Os*bZIP74*. We linked a large promoter fragment (~1.5 kb) and several truncated segments of Os*bZIP74* to the firefly luciferase reporter (Figure 4a). In dual‐luciferase reporter assays, when the truncated form of OsNTL3 (NTL3∆C) was co‐transformed with the full‐size reporter (pbZIP74‐A) in tobacco leaves, the relative luciferase activity was higher compared with that when the empty vector control was co‐transformed (Figure 4b). In contrast, the truncated forms pbZIP74‐B and pbZIP74‐C had lower reporter activity when the NTL3∆C effector was co‐transformed (Figure 4b). Further, the relative luciferase activity was increased when pbZIP74‐D and pbZIP74‐E were co‐transformed (Figure 4b). To demonstrate the direct binding of OsNTL3 to Os*bZIP74* promoter, we purified MBP‐tagged OsNTL3 (MBP‐NTL3∆C) and carried out electrophoretic mobility shift assays (EMSAs) with the biotin‐labelled Os*bZIP74* promoter DNA (98 bp within pbZIP74‐E). When MBP‐NTL3∆C was incubated with the biotin‐labelled DNA, a band shift was observed (Figure 4c), reflecting the formation of protein‐DNA complex. Adding non‐labelled cold probes (50X or 100X) could compete the binding (Figure 4c), suggesting that the binding is quite specific. Chromatin immunoprecipitation (ChIP) experiments were also carried out with myc‐OsNTL3∆C expressing plants using anti‐myc antibody. It was found that myc‐OsNTL3∆C is enriched significantly at the promoter regions of Os*bZIP74* and other OsNTL3‐dependent genes (Figure 4d). Taken together, these results demonstrated that OsNTL3 directly binds to Os*bZIP74* promoter and regulates the expression of Os*bZIP74* in rice under heat stress conditions.

### Inducible expression of Os*NTL3∆C* increases heat stress tolerance in rice

**Figure 5:**
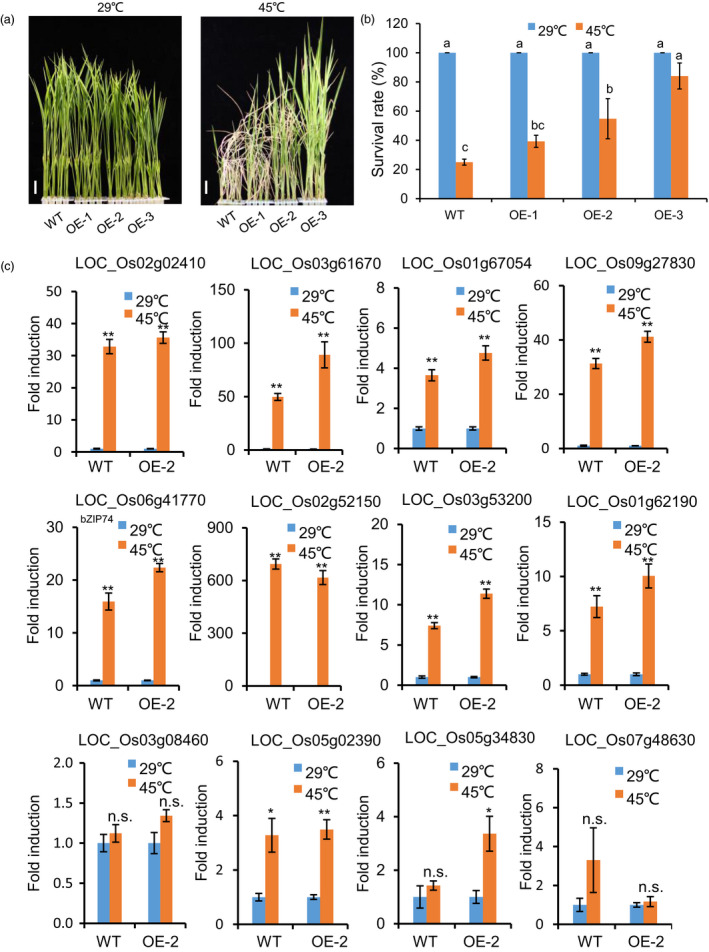
Overexpression of Os*NTL3∆C* increases heat stress tolerance in rice seedlings (a–b) Enhanced heat stress tolerance in *NTL3∆C* overexpression plants. The truncated form of NTL3 devoid of the transmembrane domain (NTL3∆C) was overexpressed with the heat inducible BiP promoters in rice. Eight‐day‐old rice seedlings grown at 29 ºC were transferred to 45 ºC for 10 days and photographed after recovering at 29 ºC for 4 days (a). Bar = 1 cm. Survival rate was count based on the appearance of newly developed green leaves (b). Totally, 84 rice plants under each temperature condition for each genotypes were examined for phenotype analysis. Different letters indicate significant differences in comparisons between two samples as determined by LSD test following ANOVA analysis (*P* < 0.05). (c) Expression of Os*NTL3*‐dependent genes under heat stress conditions in Os*NTL3∆C* overexpression (OE) plants in rice. Eight‐day‐old WT and Os*NTL3∆C* expressing plants grown at 29 ºC were treated at 45 ºC for 1 h, and total RNA was extracted for qRT‐PCR. Fold induction is the expression level of Os*NTL3* in stressed plants relative to that in non‐stressed plants, both of which were normalized to that of the internal control *ACTIN*. Error bars represent SE (*n* = 3). Asterisks indicate significance levels when comparing to the control in t‐test. (*, *P* < 0.05; **, *P* < 0.01; n.s., not significant at *P* < 0.05).

In order to know whether manipulation of Os*NTL3* could enhance heat stress tolerance in rice, we overexpressed the processed form of OsNTL3 (OsNTL3∆C) in rice (WT background) with heat inducible promoters from *BiP2* and *BiP4* and generated several transgenic rice lines. Both *BiP2* and *BiP4* are highly up‐regulated by heat stress treatment (Data S1) while transient expression experiment in tobacco leaves showed that YFP‐NTL3∆C signals were exclusively found in the nucleus (Figure S3). Under normal growth temperature conditions, total expression level of Os*NTL3* in the transgenic lines (OE‐1 to OE‐3) was similar to that in the WT plants, in contrast, under heat stress conditions, total expression level of Os*NTL3* was much higher in the transgenic lines (OE‐1 to OE‐3) than that in the WT plants (Figure S4), suggesting that the transgene Os*NTL3∆C* is induced under heat stress condition. The transgenic plants grew as normally as the WT at standard growth temperature (29 ºC); however, when plants were subjected to a period of high temperature (45 ºC) stress, the survival rate of the transgenic plants was much higher than that of the WT plants after recovery (Figure 5a‐b). We also checked the expression of above‐mentioned 12 genes in the WT and OsNTL3∆C overexpression plants under heat stress condition. Among them, seven genes encoding two calreticulin proteins, one protein disulfide isomerase, one calmodulin‐related calcium sensor protein, one C2H2 zinc finger protein, one NAC domain‐containing protein and OsbZIP74 were up‐regulated by heat stress with higher fold change in OsNTL3∆C overexpression plants than that in WT (Figure 5c). The other five genes probably requires other factors to be induced under heat stress conditions, and overexpression of *OsNTL3∆C* alone is not sufficient to induce them. Nevertheless, overexpression of the processed form of OsNTL3 more efficiently up‐regulates heat stress responsive genes and improves heat stress tolerance in rice seedlings.

### Heat‐induced expression of Os*NTL3* is dependent on OsbZIP74 in rice

**Figure 6:**
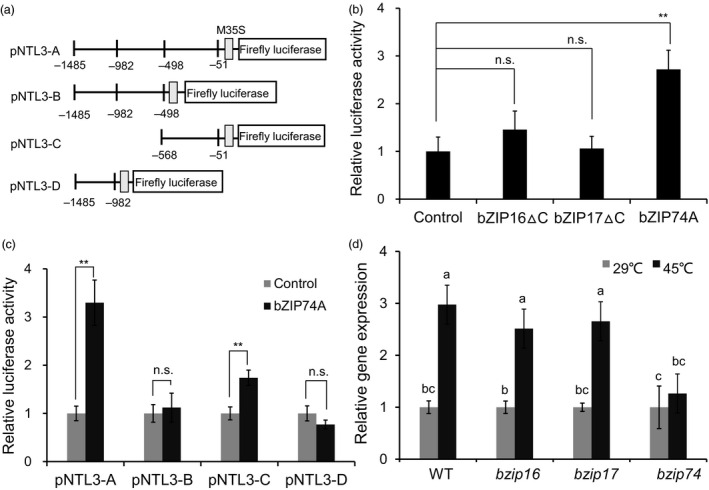
Up‐regulation of *OsNTL3* by heat stress is dependent on OsbZIP74 in rice. (a) Diagrams showing various DNA fragments of Os*NTL3* promoter linked to the minimal 35S promoter (M35S) and the reporter firefly luciferase. The position relative to the transcription start site (TSS) is indicated below. (b) Transactivation of the full‐length size promoter pNTL3‐A by OsbZIP74. The processed form of OsbZIP16 (bZIP16∆C), OsbZIP17 (bZIP17∆C) or OsbZIP74 (bZIP74A) was used as an effector in the dual‐luciferase assays. (c) Requirement of segment pNTL3‐C for the transcriptional activation. The relative luciferase activity is the firefly luciferase activity normalized to the Renilla luciferase activity driven by the 35S constitutive promoter, which was then normalized to the empty vector control. (d) OsbZIP74‐dependent up‐regulation of Os*NTL3* by heat stress. Eight‐day‐old WT and targeted‐gene‐edited mutant plants of Os*bZIP16* (*bzip16*), Os*bZIP17* (*bzip17*) and Os*bZIP74* (*bzip74*) were treated with heat stress, and Os*NTL3* expression was checked by qRT‐PCR. Relative gene expression is the expression level of Os*NTL3* in stressed plants relative to that in non‐stressed plants, both of which were normalized to that of the internal control *ACTIN*. Error bars represent SE (*n* = 3). Asterisks indicate significance levels when comparing to the control in t‐test in b–c. (*, *P* < 0.05; **, *P* < 0.01; n.s., not significant at *P* < 0.05). Different letters indicate significant differences in comparisons between two samples as determined by LSD test following ANOVA analysis (*P* < 0.05) in d. *bzip74‐1* was used in the study.

Os*NTL3* is constitutively expressed and up‐regulated by heat stress (Figure 1a). OsbZIP16, OsbZIP17 and OsbZIP74 are membrane‐associated transcription factors involved in ER stress response in rice (Liu and Howell, 2016; Lu *et al.*, 2012; Takahashi *et al.*, 2012). To further determine whether these bZIP transcription factors regulate Os*NTL3* expression under heat stress condition, we firstly linked the promoter region (~1.5 kb) of Os*NTL3* to the firefly luciferase reporter (Figure 6a). In a dual‐luciferase reporter assay system in tobacco (*Nicotiana benthamiana*) leaves, when the processed form of OsbZIP16 (bZIP16∆C) or OsbZIP17 (bZIP17∆C) was co‐transformed with the reporter, the relative luciferase activity was similar to that when the empty vector control was co‐transformed (Figure 6b). In contrast, when the activated form of OsbZIP74 (bZIP74A) was co‐transformed with the reporter, the promoter activity was significantly increased (Figure 6b). We further made three truncated versions of promoters for the reporter (Figure 6c) and performed the effector‐reporter assays. It was found that the segment C (−568 bp to −51 bp relative to TSS) had the highest relative luciferase activity (Figure 6c). These results demonstrated that OsbZIP74 is sufficient to induce the promoter activity of Os*NTL3*. We generated loss‐of‐function rice mutants of Os*bZIP16* (*bzip16*), Os*bZIP17* (*bzip17*) and Os*bZIP74* (*bzip74*) with the CRISPR‐Cas9 system in ZH11 background (Yan *et al.*, 2015) (Figure S5‐S7). The expression of Os*NTL3* was up‐regulated by heat stress in wild‐type (WT, subsp. ZH11), *bzip16* mutant as well as in *bzip17* mutant plants, in contrast, the expression of Os*NTL3* was not significantly up‐regulated in the *bzip74* mutant plants (Figure 6d). Thus, up‐regulation of Os*NTL3* by heat stress is dependent on OsbZIP74 in rice. We also checked heat stress sensitivity in the *bzip74* mutant plants. However, no significant difference was observed among WT and two *bzip74* independent mutant plants (Figure S8), suggesting that the function of OsNTL3 is not fully dependent on OsbZIP74, and OsbZIP74 has multifaceted roles in heat stress responses in rice.

## Discussion

Drought and heat stress are two major environmental stresses that threaten the sustainable crop production worldwide (Nadeem *et al.*, 2018). It has been reported that extreme heat stress weathers have caused a very large amount of grain yield loss in many regions of the world (Ciais *et al.*, 2005; He *et al.*, 2018; Smith and Katz, 2013; Yang *et al.*, 2017). Engineering heat stress tolerant crop varieties are needed for food security in future. The transcriptional regulator DNA polymerase II subunit B3‐1 (DPB3‐1) is a positive regulator of dehydration‐responsive element‐binding protein 2A (DREB2A) in *Arabidopsis* (Sato *et al.*, 2014). Overexpression of the *Arabidopsis* DPB3‐1 in rice did not affect plant growth or yield under normal growth conditions but enhanced heat stress tolerance in rice (Sato *et al.*, 2016). Interestingly, overexpression of an *Arabidopsis* receptor‐like kinase ERECTA (ER) in rice also confers thermotolerance in the greenhouse and in field tests at multiple locations in China during several seasons without growth penalty (Shen *et al.*, 2015). Thermotolerance 1 (TT1), which encodes an α2 subunit of the 26S proteasome involved in the degradation of ubiquitinated proteins, is a major quantitative trait locus (QTL) for thermotolerance in African rice (*Oryza glaberrima*). Overexpression of OgTT1 was associated with markedly enhanced thermotolerance in rice (Li *et al.*, 2015). In the current study, we found that inducible expression of an processed form of OsNTL3 enhances heat stress tolerance in rice at seedling stage (Figure 5a‐b), providing a candidate strategy for engineering or breeding thermotolerant crops in future.

Accumulation of misfolded proteins in ER leads to the ER stress response, in which membrane‐associated transcription factors (MTFs) are involved (Liu and Howell, 2010). Although AtbZIP28 and AtbZIP60 are important for maintaining fertility under heat stress conditions in *Arabidopsis* (Zhang *et al.*, 2017), increasing heat tolerance in plants by directly manipulating the expression levels of At*bZIP28* and/or At*bZIP60* might not be practical. For example, overexpression of the activated forms of AtbZIP17, AtbZIP28, or AtbZIP60 in Arabidopsis has great inhibitory effects on plant growth and development, (Iwata *et al.*, 2008; Liu *et al.*, 2007; Liu *et al.*, 2008). Further, some of the downstream genes of AtbZIP28 or AtbZIP60 are involved in programmed cell death (Yang *et al.*, 2014b). Here in the current paper, we demonstrated that inducible expression of OsNTL3 devoid of the transmembrane domain could enhance heat stress tolerance in rice at seedling stage, with no obvious defective effects on plant overall growth (Figure 5a‐b). Therefore, OsNTL3 might be an ideal target for breeding more heat tolerant rice variety in future.

In *Arabidopsis*, two membrane‐associated NAC transcription factors are involved in UPR, with AtNAC062 promoting cell survival while AtNAC089 inducing cell death under ER stress conditions (Yang *et al.*, 2014a; Yang *et al.*, 2014b). In the current study, mutation of Os*NTL3* in rice confers heat stress sensitivity (Figure 1d‐e), suggesting that OsNTL3 has a positive effect on promoting cell survival in rice under ER stress conditions. AtNAC062/AtNTL6 is induced by cold and mediates cold‐induced pathogen responses (Seo et al., 2010); however, OsNTL3 is not induced by cold treatment but by heat stress (Figure 1a). Such difference might be explained by different growth temperature habitat of Arabidopsis and rice. Regulated intramembrane proteolysis and unconventional splicing are two major ways to activate membrane‐associated bZIP family transcription factors in plants (Liu and Howell, 2016). Although how membrane‐associated NAC transcription factors are activated in plants remains enigmatic, the processing of these membrane‐associated transcription factors have been reported (Kim *et al.*, 2007; Yang *et al.*, 2014a; Yang *et al.*, 2014b). In the current study, the processed form of OsNTL3 was detected under heat and ER stress conditions by Western blotting (Figure 2b). Relocation of OsNTL3 from plasma membrane to nucleus in response to heat and ER stresses was also observed (Figure 2a), suggesting that OsNTL3 is activated under these stress conditions. OsNTL3 has transcriptional activation activity (Figure S2) and regulates several heat stress responsive genes (Figure 3), further supporting that OsNTL3 is important for heat stress tolerance in rice. It is known that heat stress denatures proteins; therefore, heat stress is assumed to cause the accumulation of unfolded/misfolded proteins in ER, eliciting the ER stress response (UPR). Os*NTL3* is up‐regulated by ER stress (Figure 1a), and many OsNTL3‐dependent heat stress genes are involved in UPR (Figure 3c). Thus, taken together, these results demonstrated that OsNTL3 mediates ER stress response under heat stress conditions.

**Figure 7:**
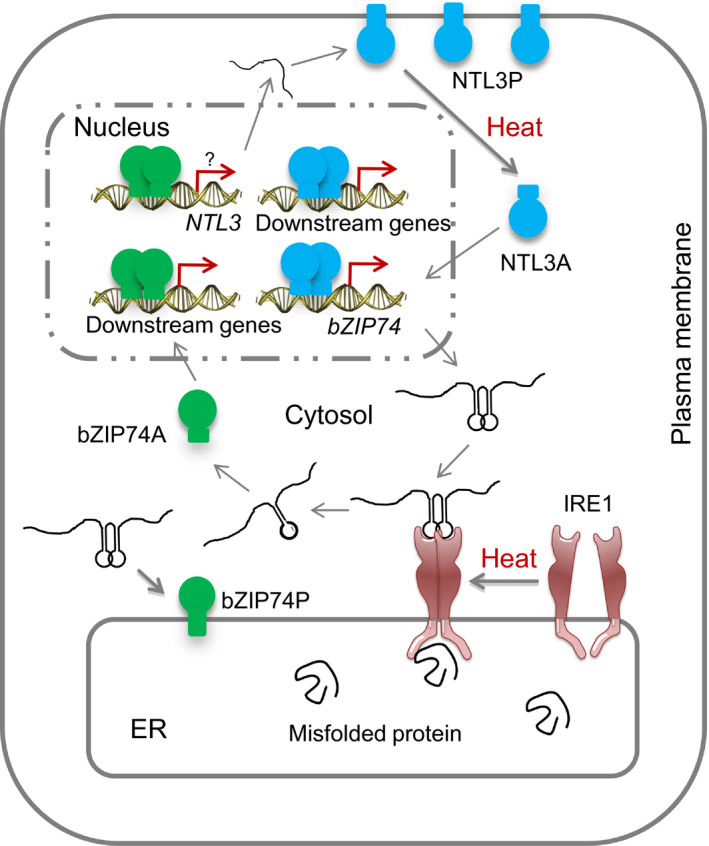
A hypothetical working model for the role of OsNTL3 in heat‐induced ER stress response in rice. Under normal growth condition, the OsbZIP74 precursor protein (bZIP74P) localizes in ER membrane while the OsNTL3 precursor protein (NTL3P) is found in plasma membrane. In response to heat stress, accumulation of misfolded proteins in ER leads to activation of the ER‐localized OsIRE1, which has both protein kinase and endonuclease domains. Twenty nucleotides in the double‐stem structure of Os*bZIP74* mRNA are spliced out and an open reading frame shift produces an activated form of OsbZIP74 (bZIP74A), which is dependent on OsIRE1. bZIP74A enters nucleus and either directly or indirectly up‐regulates Os*NTL3* and other downstream genes. Meanwhile, NTL3P is processed by an unknown mechanism, and the activated OsNTL3 (NTL3A) relocated from plasma membrane to nucleus and up‐regulates Os*bZIP74* and other downstream genes. Genetic experiments demonstrate that OsNTL3 is required for thermotolerance in rice. Thus, OsNTL3 relays heat stress signal from ER and plasma membrane to nucleus and regulates heat stress responsive genes in rice.

The activated form of OsbZIP74 were able to activate the promoter of Os*bZIP74* in an effector‐reporter assay in rice protoplasts (Hayashi *et al.*, 2012). In our studies, we found that mutation of Os*NTL3* impaired the up‐regulation of Os*bZIP74* under heat stress conditions (Figure 3c), and OsNTL3 binds to Os*bZIP74* promoter and directly regulates the promoter activity of Os*bZIP74* (Figure 4). Although the direct binding of OsbZIP74 to Os*NTL3* promoter has not yet been demonstrated, given that heat stress up‐regulates Os*NTL3* expression in an OsbZIP74‐dependent manner, and OsbZIP74 activates the promoter of Os*NTL3* in transient expression assays (Figure 6), our results suggested that OsbZIP74 and OsNTL3 form a regulatory circuit in heat stress response in rice, although both OsbZIP74 and OsNTL3 have other target genes (Figure 7).

Membrane fluidity is largely dependent on the composition of lipid molecular species, the degree of membrane saturation and different membrane proteins inside the membranes. High temperature stress immediately affects membrane fluidity and composition in plants, and studies with modification of membrane fluidity suggest that membrane fluidity plays a central role in sensing temperature stresses (Horvath *et al.*, 1998; Hugly *et al.*, 1989; Murakami *et al.*, 2000; Orvar *et al.*, 2000). Since OsNTL3 precursor protein is constitutively observed in plasma membranes, it may sense the membrane fluidity/integrity change during heat stress response and transduce the stress signals to nucleus. Interestingly, OsbZIP74 precursor protein localizes in ER, and the activation of OsbZIP74 is dependent on an ER‐localized OsIRE1 (Lu *et al.*, 2012). The ER is not only important for protein biosynthesis, but also for the synthesis of lipids and sterols essential for membrane formations in plants. Given that the expression of Os*NTL3* is regulated by OsbZIP74 under heat stress condition, heat stress effects may also be sensed at ER membrane and relayed to OsNTL3 at plasma membrane. Therefore, OsbZIP74 and OsNTL3 are important for coordinating the functions of ER and plasma membrane under heat stress conditions in rice, through regulating downstream genes in the nucleus after activation (Figure 7).

## Experimental procedures

### Plant materials and growth conditions

All rice materials are in subspecies Nipponbare background unless mentioned in the text. Rice seeds were germinated in water at 37 ºC in darkness for 2 days. Germinated seeds were placed into 96‐well plates and grown in Kimura B nutrient solution under 20 000 lux white fluorescent light (13 h light/ 11 h dark) at 29 ºC and with 65% relative humidity. 8‐day‐old seedlings were used for various stress treatments. Rice seedlings were treated for 4 hr with specific concentration of reagents as following: 2 mm DTT, 5 μg/mL TM, 15% PEG 4000, 250 mm NaCl and 0.1 mm ABA. For heat and cold stress treatment for gene expression analysis, rice seedlings were immerged for 4 hr in water pre‐warmed or pre‐chilled to 45 ºC or 4 ºC, respectively. Roots were harvested for RNA extraction and subsequent gene expression analysis.

### Transgenic overexpression and targeted gene editing

For inducible expression of the processed form of OsNTL3, fragments of the rice *BiP2* (LOC_Os03g50250) and *BiP4* (LOC_Os05g35400) promoter sequences (1642 bp and 2232 bp upstream sequences relative to the ATG start codon, respectively) were amplified from Nipponbare genomic DNA and cloned into the plant binary vector pCAMBIA 1300 with the *EcoRI* and *KpnI* restriction cutting sites. Subsequently, the nucleotide sequences (996 bp in length) encoding the truncated OsNTL3 (LOC_Os01g15640) were amplified from Nipponbare and inserted into the vectors with *SalI* and *PstI* sites to generate the expression vectors pBiP2::OsNTL3∆C or pBiP4::OsNTL3∆C. For conditional expression of the truncated form of MYC‐tagged OsNTL3, the coding sequence of OsNTL3 was amplified with PCR and inserted into pER8 after digestion with *XhoI* and *XbaI*/*SpeI* restriction enzymes to produce the pER8::MYC‐NTL3∆C construct. For over expression of the full‐length of GFP‐tagged OsNTL3, the coding sequence of OsNTL3 and green fluorescence protein (GFP) tag were amplified with PCR and inserted into pCAMBIA1301 to produce the vector ProUbiqutin::GFP‐OsNTL3 constructs. For generation of gene‐edited plants using CRISPR/Cas9 technology, gene‐specific guide sequences (sgRNAs) were designed and the expression cassettes were cloned into pCAMBIA1300 (Feng *et al.*, 2013). Error‐free constructs were introduced into plants by agro‐bacterial mediated stable transformation. Transgenic plants expressing pBiP2::OsNTL3∆C (OE‐1) or pBiP4::OsNTL3∆C (OE‐2 and OE‐3) were used for phenotypic analysis. All the primers are listed in Table S1.

### Physiological parameter determination

For detection of H_2_O_2_, rice leaves of four‐week‐old wild‐type and mutant plants were immersed in 1 mg/mL 3, 3‐diaminobenzidine (DAB) for 10 min with vacuum applied and then for 24 hr in darkness. Subsequently, the treated leaves were decolorized in 80% ethanol and then photographed. For the electrolytic leakage assay, rice leaves were placed in 20 ml of deionized water in a beaker for 2 hr at room temperature, and the initial conductivity (E1) was recorded using a microprocessor based conductivity meter (Model 1601, ESICO). The beaker was then placed on electromagnetic oven for boiling for 10 min to release all the electrolytes into the solution, cooled to room temperature and the final conductivity (E2) was recorded. The electrolytic leakage was calculated as the ratio of conductivity before boiling to that after boiling (E1/E2).

### Effector‐reporter assays

For dual‐luciferase activity assay, fragments of Os*NTL3* promoter or Os*bZIP74* promoter were synthesized and inserted into pGreen0800‐II with the 35S minimal promoter included to generate the reporter vector. The coding sequence (1–996 bp) of Os*NTL3* was inserted into the pCAMBIA1300‐35S vector to generate the effector vector 35S::NTL3∆C. The coding sequences of the activated forms of OsbZIP16 and OsbZIP17 and the spliced form of Os*bZIP74* were inserted into the pGreenII 62‐SK vector to generate the effector vector 35S::bZIP16∆C, 35S::bZIP17∆C and 35S::bZIP74A, respectively. Error‐free constructs were transiently expressed in tobacco (*Nicotiana benthamiana*) leaves via *Agrobacterium tumefaciens* strain GV3101. The infiltrated plants were grown in a greenhouse for an additional 3 days, and the luciferase activities were measured with a dual‐luciferase reporter assay kit (Promega). All the primers are listed in Table S1.

### Protein subcellular localization analysis

For protein subcellular localization studies, the coding sequence of full‐length OsNTL3 was fused to GFP and GFP‐NTL3 was constitutively expressed in stably transformed rice plants driven by the Ubiquitin promoter. Transgenic rice grown at 29 ºC were transferred to either 45 ºC or kept at 29 ºC for 2 hr, or treated with 5 μg/ml TM and 2 mm DTT for 2 hr, and root tips were observed under a confocal microscopy (Zeiss LSM A710). YFP‐NTL3∆C was also constructed and transiently expressed in tobacco (*Nicotiana benthamiana*) leaves via *Agrobacterium‐*mediated transformation for subcellular localization. PI staining and DAPI staining were used to highlight the cell membrane and nucleus, respectively. All the primers are listed in Table S1.

### Western blotting analysis

For Western blotting analysis of the processing of GFP‐OsNTL3 in plants, total proteins were extracted with the extraction buffer (125 mm Tris‐HCl, pH 8.0, 375 mm NaCl, 2.5 mm EDTA, 1% SDS and 1% β‐mercaptoethanol) from the transgenic rice plants, and protein concentration was determined by a BCA Protein Assay Kit (Solarbio, Beijing, China). After that, proteins were separated in 10% SDS‐PAGE gels and analysed using anti‐GFP antibody. Anti‐histone H3 was used for a loading control.

### RNA‐Seq analysis

Approximately eight days after germination, rice seedlings were transferred to 45 ºC, or kept at 29 ºC. After 3 hr, seedlings were collected for each of the three replicates and immediately frozen in liquid nitrogen. Total RNA was extracted with TRIZOL (Invitrogen). The cDNA libraries were constructed following Illumina standard protocols and sequenced on the Illumina HiSeq 4000 platform (Major Bio). RNA‐seq reads were aligned to the rice reference genome (http://rice.plantbiology.msu.edu/index.shtml) using TopHat after filtering out low‐quality (lowest base score < 20) reads using SeqPrep and Sickle (Trapnell *et al.*, 2012). Gene expression level was calculated and normalized to FPKM (fragments per kilobase of transcript per million mapped reads) with RSEM (Li and Dewey, 2011). Differential gene expression was determined using the R package edgeR (Anders and Huber, 2010; Robinson *et al.*, 2010). The cut‐off for significant differential expression was set as log2 (fold change) ≥1 and false discovery rate < 0.05. The RNA‐Seq data from this article can be found in the Gene Expression Omnibus (GEO) under accession number GSE122021.

### qRT–PCR and chromatin immunoprecipitation (ChIP)‐qPCR analysis

Total RNA was extracted as described in the text using a RAN Prep Pure Plant Kit (Tiangen, Beijing, China). For reverse transcription, 2 μg of RNA and oligo (dT) primers were used to synthesize cDNA in a 20 μL reaction using the M‐MLV reverse transcriptase (Takara, Beijing, China). Quantitative RT‐PCR (q‐PCR) was performed using the SuperReal PreMix Color (Tiangen) in the CFX96 real‐time system (Bio‐Rad, Hercules, CA, USA). The gene expression levels in three biological replicates were calculated using the ∆∆Ct (threshold cycle) method. *ACTIN* was used as an internal control for normalization. ChIP was performed according to the standard protocols. Four‐week‐old WT and pXVE::myc‐NTL3△C transgenic seedlings were treated with 10 mM beta‐estrodial for 16 hr. One gram of plants was collected for ChIP assays. Protein A agarose (Millipore, Temecula, CA, USA) and myc (Abmart) were used for immunoprecipitation. About 10% of sonicated but nonimmunoprecipitated chromatin was used as an input DNA control. Precipitated DNA and input DNA were analysed by quantitative PCR. Student’s t‐test was used to evaluate the significance of differences between samples. All the primers are listed in Table S1.

### Transcriptional activation activity assay

For transcriptional activation activity assay in yeast, various truncated forms of OsNTL3 were inserted into pGBKT7 (Clontech). Plasmids were transferred to yeast strain AH109 (Clotech) according to a commercial kit (ZYMO RESEARCH) and cultured on selective plates at 30 ºC for 3–5 days. Transcriptional activation activity was evaluated based on the activation of the *HIS4* reporter. All the primers are listed in Table S1.

### Electrophoretic mobility shift assay (EMSA)

The Os*bZIP74* probe was created by annealing together the complementing oligonucleotides and biotinylated with the Biotin 3’‐end DNA Labelling Kit (Thermo Fisher Scientific, Waltham, MA, USA). MBP‐NTL3∆C proteins were expressed in *E. coli* strain BL21 and purified by amylose agarose beads. EMSA was performed using a LightShift Chemiluminescent EMSA Kit (Thermo Fisher Scientific), according to the manufacturer’s protocols. Briefly, each 20 μl binding reaction contained 2 μl binding buffer, 0.3 μl Poly (dI‐dC), 4 μg purified protein, 50 nmol biotin‐labelled probe or certain amount of unlabelled probe as the competitor. The binding reactions were allowed to incubate at room temperature for 20 min and run on a 5% non‐denaturing polyacrylamide gel. The complex was transferred to a nylon membrane (GE). After UV light cross‐linking, the DNA on the membrane was detected with the Chemiluminescent Nucleic Acid Detection Module (Thermo Fisher Scientific).

## Author contributions

X.H.L. and Y.S.L. performed the experiments; J.X.L., X.H.L and S.J.L. designed the experiments; J.X.L. and X.H.L. analysed the data; W. Y. and Z.T.Y. contributed to plant materials and constructs; X.H.L. and J.X.L. wrote the paper.

## Conflict of interest

The authors declare no conflicts of interest.

## Supporting information


**Figure S1** Characterization of mutation in OsNTL3.
**Figure S2** OsNTL3 has transcriptional activation activity.
**Figure S3** Subcellular localizations of YFP‐OsNTL3∆C.
**Figure S4** Validation of transgenic expression.
**Figure S5** Characterization of mutation in *O*s*bZIP16*.
**Figure S6** Characterization of mutation in *bZIP17*.
**Figure S7** Characterization of mutation in *O*s*bZIP74*.
**Figure S8** Loss‐of‐function of OsbZIP74 does not affect heat stress sensitivity in rice.

**Table S1** Primers used in this study.

**Data S1** Differential expressed heat stress responsive genes in rice seedlings.
