# Supplementary material for: A membrane‐associated NAC transcription factor OsNTL3 is involved in thermotolerance in rice
Source: Plant Biotechnol J. 2019 Dec 6;18(5):1317–29. doi: 10.1111/pbi.13297 (PMC7152603; doi:10.1111/pbi.13297)
Supplement: Supplementary file 1 — Figure S1 Characterization of mutation in OsNTL3. Figure S2 OsNTL3 has transcriptional activation activity. Figure S3 Subcellular localizations of YFP‐OsNTL3∆C. Figure S4 Validation of transgenic expression. Figure S5 Characterization of mutation in OsbZIP16. Figure S6 Characterization of mutation in bZIP17. Figure S7 Characterization of mutation in OsbZIP74. Figure S8 Loss‐of‐function of OsbZIP74 does not affect heat stress sensitivity in rice. [file PBI-18-1317-s001.pdf]

LOC_Os01g15640.1(NTL3) MESLRDMVLPPGFGFHPKDTELISHYLKKKIHGQKIEYEIIPEVDIYKHEPWDLPAKCDV
LOC_Os01g15640.1(*ntl3-1*) MESLRDMVLPPGFGFHPKDTELISHYLKKKIHGQKIEYEIIPEVDIYKHETMGFTCKVRC
LOC_Os01g15640.1(*ntl3-2*) MESLRDMVLPPGFGFHPKDTELISHYLKKKIHGQKIEYEIIPEVDIYKHEPWDLPAKCDV
****

LOC_Os01g15640.1(NTL3) PTQDNKWHFFAARDRKYPNGSRSNRATVAGYWKSTGKDRAIKMGKQTIGTKKTLVFHEGR
LOC_Os01g15640.1(*ntl3-1*) SNSG--
LOC_Os01g15640.1(*ntl3-2*) PTQDNKWHFFAARDRKYPNGSRSNRATVAGYWKSTGKDRAIKMGKQTIGTKKTLVFHEGR

LOC_Os01g15640.1(NTL3) PPTGRRTEWIMHEYYIDERECQACPDMKDAYVLCRITKRNDWIPGNGNELDNSDPHPEPY
LOC_Os01g15640.1(*ntl3-1*) --
LOC_Os01g15640.1(*ntl3-2*) PPTGRRTEWIMHEYYIDERECQACPDMKDAYVLCRITKRNDWIPGNGNELDNSDPHPEPY

LOC_Os01g15640.1(NTL3) DAPPSVISTEQLNPAAEPVVGVEAAPVTVAEPDGVTTSAITANIPSPSDDINLDDWLNEL
LOC_Os01g15640.1(*ntl3-1*) --
LOC_Os01g15640.1(*ntl3-2*) DAPPSVISTEQLNPAAEPVVGVEAAPVTVAEPDGVTTSAITANIPSPSDDINLDDWLNEL

LOC_Os01g15640.1(NTL3) FDPFFDPEQSLASADLSPDEQNVESSNVGALAPKVEQDYSSPNENVVDDTEYLLPEDVYN
LOC_Os01g15640.1(*ntl3-1*) --
LOC_Os01g15640.1(*ntl3-2*) FDPFFDPEQSLASADLSPDEQNVESSNVGALAPKVEQDYSSPNENVVDDTEYLLPEDVYN

LOC_Os01g15640.1(NTL3) ILHPGTDDFNMLQNPLDQYPIQYATDVWSGIQKEELWSPQANAEPSQSNEAADNGIIRRY
LOC_Os01g15640.1(*ntl3-1*) ---
LOC_Os01g15640.1(*ntl3-2*) ILHPGTDDFNMLQNPLDQYPIQYATDVWSGIQKEELWSPQANAEPSQSNEAADNGIIRRY

LOC_Os01g15640.1(NTL3) RSMKTPETSVPQFKGKTQAKMRVGINKMATSSSESINQTIKFENSGRLVEHQKNQAHDVA
LOC_Os01g15640.1(*ntl3-1*) --
LOC_Os01g15640.1(*ntl3-2*) RSMKTPETSVPQFKGKTQAKMRVGINKMATSSSESINQTIKFENSGRLVEHQKNQAHDVA

LOC_Os01g15640.1(NTL3) STKRSDAGKPSTELSSNRGFLRGIRNAFAGCSDARWNMILVAGFAIGVAVVALHIGQRLG
LOC_Os01g15640.1(*ntl3-1*) --
LOC_Os01g15640.1(*ntl3-2*) STKRSDAGKPSTELSSNRGFLRGIRNAFAGCSDSMEHDTCCGFRYWSRCGSASYRPTPWI
. .

LOC_Os01g15640.1(NTL3) LSQRDQQHT---
LOC_Os01g15640.1(*ntl3-1*) --
LOC_Os01g15640.1(*ntl3-2*) KPERSAAYLAFRFAGYWVSMLFAIWRNVNKLRSLVGAEHLMYAPFFLCWFCTYYYLRFWS

LOC_Os01g15640.1(NTL3) ------
LOC_Os01g15640.1(*ntl3-1*) ------
LOC_Os01g15640.1(*ntl3-2*) TFVLAS

Figure S1. Characterization of mutation in OsNTL3. Protein sequences of OsNTL3 in WT (NTL3) and mutants (*ntl3-1* and *ntl3-2*) derived from the CRISPR-CAS9 technology. The NAC DNA-binding domain is shown in red. The amino acids for a new C-terminus of NTL3 in *ntl3-2* mutant is boxed.

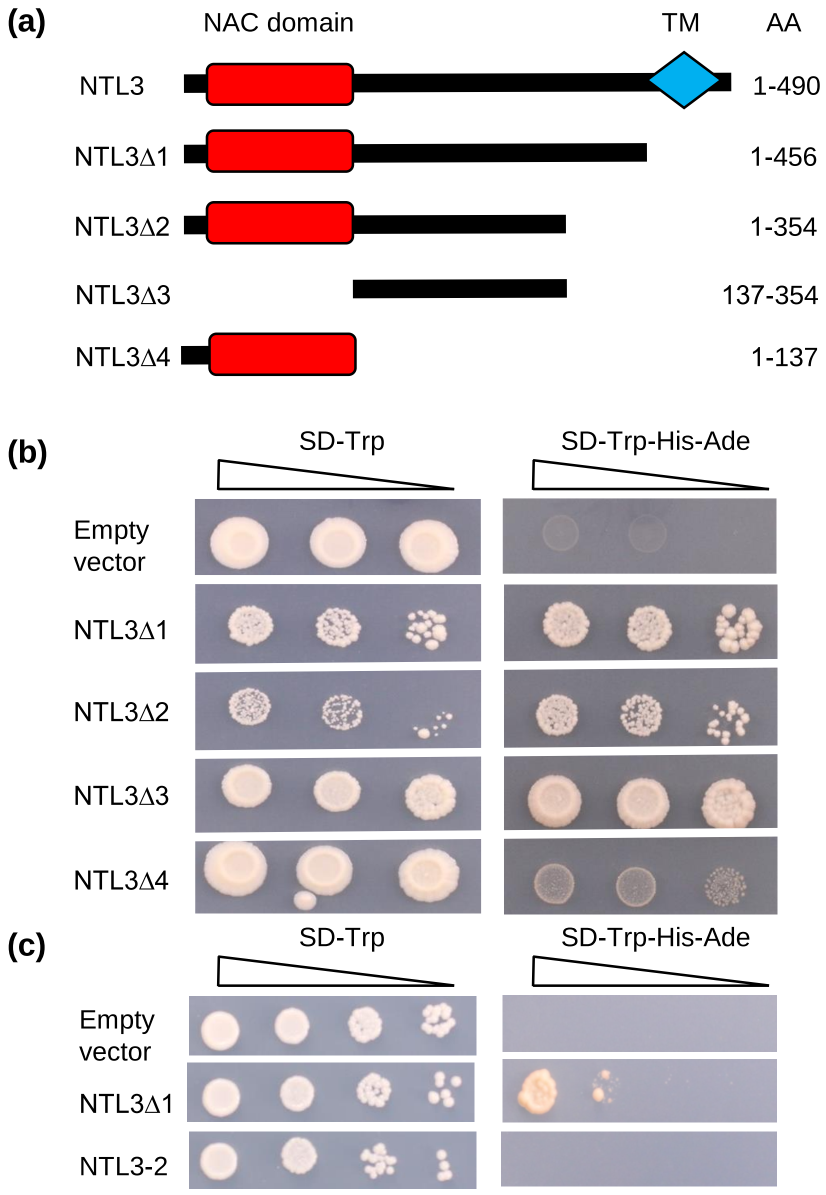

Figure S2. OsNTL3 has transcriptional activation activity. (a) Diagram showing various segments of OsNTL3 that were fused to the yeast GAL4 DNA-binding domain. The NAC domain and transmembrane domain (TM) were shown in rectangle and diamond, respectively. Amino acid (AA) positions were indicated for each segment. (b-c) Activation of the *His* and *Ade* reporter genes in yeast cells. Three different dilutions of yeast cells were spotted on nutritional selection medium. The mutated form of OsNTL3 (NTL3-2) was obtained from the *ntl3-2* rice mutant plants and used for comparison (c).

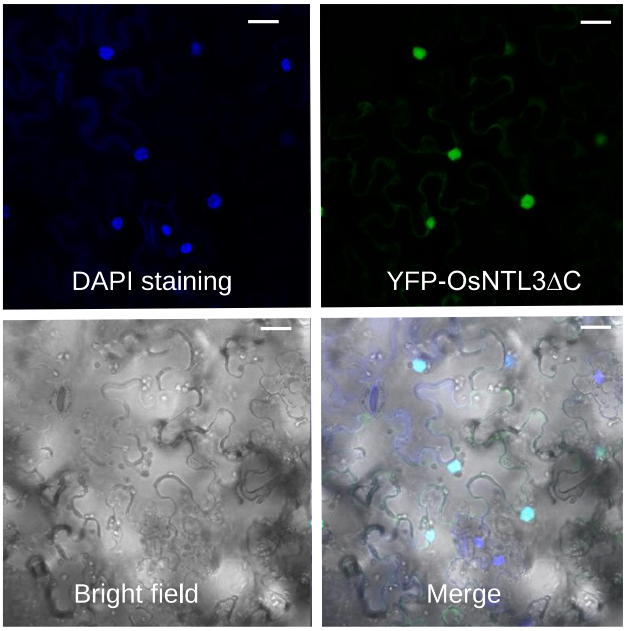

Figure S3. Subcellular localizations of YFP-OsNTL3ΔC. The YFP-tagged truncated OsNTL3 devoid of the transmembrane domain were transiently expressed in tobacco leaves. DAPI staining was used to visualize the nuclei. Bar = 50 μm.

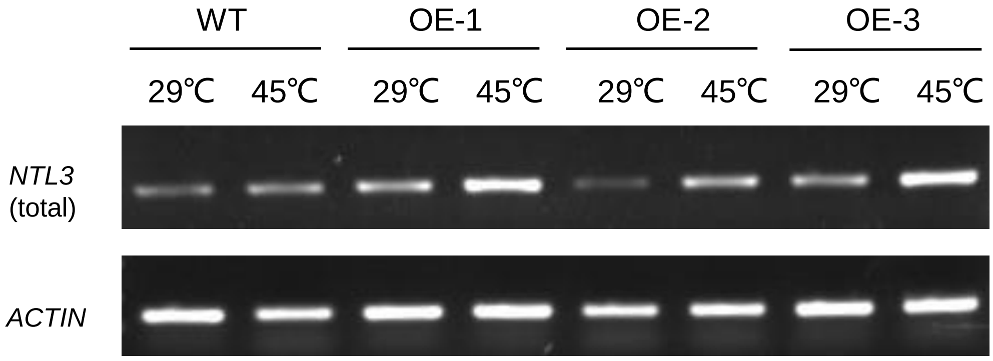

Figure S4. Validation of transgenic expression. Total RNA were extracted from the wild-type (WT) control plants and NTL3ΔC overexpression (OE1-3) plants and the expression of total Os*NTL3* was detected by RT-PCR. *ACTIN* was used as an internal control.

```
LOC_Os07g44950.1(bZIP16)   MAEPDLLAPFADLPFPPGDDFPDFPTLGDDAFALEDFDLDDLDFDFDVDLFPPDAPPPVT
LOC_Os07g44950.1(bzip16)   MAEPDLLAPFADLPFPPGDDFPDFPTGMTPSRWRISISTIWTSTSMWISSRRMRRRR---
                           **************************
LOC_Os07g44950.1(bZIP16)   TSSSSSAAGSPEAGTSSAGDGGSKNEESADSSSPSRSGSDGGGGKDGKDDEAKRRARLVR
LOC_Os07g44950.1(bzip16)   ------------------------------------------------------------

LOC_Os07g44950.1(bZIP16)   NRESAHQSRQRKKQYVEELEGKVKVMQATIADLTARISCVTAENAALKQQLGGAAGAGAA
LOC_Os07g44950.1(bzip16)   ------------------------------------------------------------

LOC_Os07g44950.1(bZIP16)   APPPPMPMYPAVYPLPMPWIHPAYAMRGSQVPLVPIPRLKTQQPASTPEPPAKKARKTKK
LOC_Os07g44950.1(bzip16)   ------------------------------------------------------------

LOC_Os07g44950.1(bZIP16)   VAGVSLLGLLFLMMVCGCLVPAVNRMYGAAYTGEGAAIVPSHHGRILAVEGPQNSVSNGV
LOC_Os07g44950.1(bzip16)   ------------------------------------------------------------

LOC_Os07g44950.1(bZIP16)   DPKVPQNGSETLPALLYLPRNGKHVKINGNLVIKSIVASEKASSRLSNYGEKGSGNQGKE
LOC_Os07g44950.1(bzip16)   ------------------------------------------------------------

LOC_Os07g44950.1(bZIP16)   ETSLAIPGYVAPLEAGEVMDSAKGMNELMALAPGDGSIYREDDGMLPQWFSEAMSGPMLN
LOC_Os07g44950.1(bzip16)   ------------------------------------------------------------

LOC_Os07g44950.1(bZIP16)   SGMCTEVFQFDLSPTTADANGIVPVYSGSVTNTSQNYTENLPSGPVQKVKNRRISYSEAI
LOC_Os07g44950.1(bzip16)   ------------------------------------------------------------

LOC_Os07g44950.1(bZIP16)   PLRGSTSNDTDHFKAPPKNHSQSHAGRKPVSSVVVSVLADPREASDRDGEGRISSNSLSR
LOC_Os07g44950.1(bzip16)   ------------------------------------------------------------

LOC_Os07g44950.1(bZIP16)   IFVVVLIDSVKYVTYSCVLPFKSHSPHL
LOC_Os07g44950.1(bzip16)   ----------------------------
```

Figure S5. Characterization of mutation in Os*bZIP16*. Protein sequences of OsbZIP16 in WT (bZIP16) and mutant (*bzip16*) derived from the CRISPR-CAS9 technology are aligned. The bZIP DNA-binding domain is shown in red.

```
LOC_Os05g34050.1(bZIP17)  MAEPALLDPTAAFDLRLYPAHLFDHELPLAGGGGGDDDDDLPLDGLEFDLPGDFSVEDFL
LOC_Os05g34050.1(bzip17)  MAEPALLDPTAAFDLRLYPAHLFDHELPLAGGGGGDDDDDLPLTGSSSTCPAISPWRTSS
                          ******************************************* *     *
LOC_Os05g34050.1(bZIP17)  LRSPERDDSGEGSAAGSGPTASPSSSPTTSASNSAVANGSGGEVKHEESDEGRSGGGDPK
LOC_Os05g34050.1(bzip17)  SGLRSGTTPARALLPDPGPPPPRRRPRPPRRPTPPSPTAAAARSSTRSRMRGGAVGVTPS
                                           **                                *   *  *
LOC_Os05g34050.1(bZIP17)  WSLKRKQASPGPSSDAAKCRRSGDGDVSPSASASRTAVDSDEGGTVCEEEEDERRAARLM
LOC_Os05g34050.1(bzip17)  GA----------------------------------------------------------
                      -
LOC_Os05g34050.1(bZIP17)  RNRESAQLSRQRKKRYVEELEEKVKSMHSVINDLNSRISFVVAENATLRQQLSGGSVNCP
LOC_Os05g34050.1(bzip17)  ------------------------------------------------------------

LOC_Os05g34050.1(bZIP17)  PPGVYPPAPIPGMHFPWMPGYAMRPPGSHVPLVPIPRLKPQQPVPSSKVVKKPESKKTVE
LOC_Os05g34050.1(bzip17)  ------------------------------------------------------------

LOC_Os05g34050.1(bZIP17)  NKSKSKTKTKKVASVSLLGLLLIMLVFGAFIPGFNHNFGMCGQSDNAMFRNFGQSHARVL
LOC_Os05g34050.1(bzip17)  ------------------------------------------------------------

LOC_Os05g34050.1(bZIP17)  SVSSQDKSSLNNSDMIGVDVGKMTGNTDGPGKKHQPAHNSSEILPALLYVPRNGKHVKIN
LOC_Os05g34050.1(bzip17)  ------------------------------------------------------------

LOC_Os05g34050.1(bZIP17)  GNLIIHSVLASEKAVAHKASKDDSDQSARDHKETSVAIARYLSLPGKDVNRQETSSADGP
LOC_Os05g34050.1(bzip17)  ------------------------------------------------------------

LOC_Os05g34050.1(bZIP17)  LPQWFREGMEGPILNSGMCSEVFQFDISTASSNPGGIIPASPVVNSSSVNATEKIPAHSA
LOC_Os05g34050.1(bzip17)  ------------------------------------------------------------

LOC_Os05g34050.1(bZIP17)  AYHGKLKNRRVMYNEAIPLTGKTANNTEPFNRTSESSSKLPDSKPASSVVVSVLADPREA
LOC_Os05g34050.1(bzip17)  ------------------------------------------------------------

LOC_Os05g34050.1(bZIP17)  GNGDGDPRVSPKPLSKIFVVVLVDGVRYVTYSCTLPFKSSSPHLVN
LOC_Os05g34050.1(bzip17)  ----------------------------------------------
```

Figure S6. Characterization of mutation in *bZIP17*. Protein sequences of bZIP17 in WT (bZIP17) and mutant (*bzip17*) derived from the CRISPR-CAS9 technology are aligned. The bZIP DNA-binding domain is shown in red.

```
LOC_Os06g41770.1(bZIP74)   MDVEFFADLDLDALLASFSSSAAAAGSGVSGLFAPSPPHDAEAGSPESVSSRRPSPSREA
LOC_Os06g41770.1(bzip74-1) MDVEFFADLDLDALLASFSSSAAAAGSGVSGLFAPSPPHDAEAGSPESVSSRRPSPSREA
LOC_Os06g41770.1(bzip74-2) MDVEFFADLDLDALLASFSSSAAAAGSGVSGLFAPSPPHDAEAGSPESVSSRRPSPSREA
                           ************************************************************
LOC_Os06g41770.1(bZIP74)   ALSEIERFLMEEGPAAEEGVGAEDFFDALLVDGGEEEEEEEGKGSEAGGSTDGDSGKEN
LOC_Os06g41770.1(bzip74-1) ALSEIERFLIGGPRGGGGGRRGGFLRRAARRRRGGGGGRRGEGE---------------
LOC_Os06g41770.1(bzip74-2) ALSEIERAPRRRRGSARRISSTRCSSTAGRRRRKKRGRGVRRGEARMGIPGRRMRWLPR
                           *******                    *
LOC_Os06g41770.1(bZIP74)   EVATPDAEKEDVEAEVDGDDPMSKKKRRQMRNRDSAMKSRERKKMYVKDLETKSKYLEAE
LOC_Os06g41770.1(bzip74-1) ------------------------------------------------------------
LOC_Os06g41770.1(bzip74-2) TRRRRMWRRRWMAMIP--------------------------------------------

LOC_Os06g41770.1(bZIP74)   CRRLSYALQCCAAENMALRQSLLKDRPVGAATAMQESAVLTETLPLVSLLWLVSIVCLLP
LOC_Os06g41770.1(bzip74-1) ------------------------------------------------------------
LOC_Os06g41770.1(bzip74-2) ------------------------------------------------------------

LOC_Os06g41770.1(bZIP74)   VPGLPNRNPVARSSAGRDLATVTGKKTSSEQQLEETLLLHGRRCKGSRARIKLDTGPFRL
LOC_Os06g41770.1(bzip74-1) ------------------------------------------------------------
LOC_Os06g41770.1(bzip74-2) ------------------------------------------------------------

LOC_Os06g41770.1(bZIP74)   AAAAC
LOC_Os06g41770.1(bzip74-1) -----
LOC_Os06g41770.1(bzip74-2) -----
```

Figure S7. Characterization of mutation in Os*bZIP74*. Protein sequences of OsbZIP74 in WT (bZIP74) and mutants (*bzip74-1* and *bzip74-2*) derived from the CRISPR-CAS9 technology are aligned. The bZIP DNA-binding domain is shown in red.

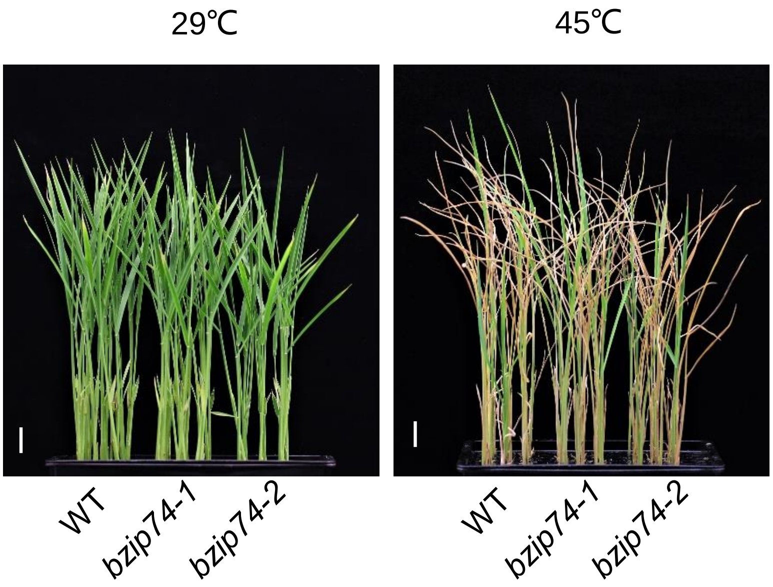

Figure S8. Loss-of-function of Os*bZIP74* does not affect heat stress sensitivity in rice. Eight-day old wild-type (WT) seedlings and two lines of targeted-gene-edited Os*bZIP74* (*bzip74-1* and *bzip74-2*) mutant seedlings grown at 29ºC were transferred to 45ºC for 4-5 days and then photographed after recovering at 29ºC for 7 days. Bar = 1 cm.
