# Supplementary material for: A membrane‐associated NAC transcription factor OsNTL3 is involved in thermotolerance in rice
Source: Plant Biotechnol J. 2019 Dec 6;18(5):1317–29. doi: 10.1111/pbi.13297 (PMC7152603; doi:10.1111/pbi.13297)
Supplement: Supplementary file 2 — Table S1 Primers used in this study. [file PBI-18-1317-s003.pdf]

Table S1. Primers Used in This Study.

Name	Locus	Forward primer (5'-3')	Reverse primer (5'-3')	Purpose
Pro:BIP2	LOC_Os03g50250	CCGGAATTCATATTCGGCCTCGTCGCCTCGC	CGGGGTACCGTTTCTTGCTCTGCTCATGCA	Over-expression
Pro:BIP4	LOC_Os05g35400	GAATTCGACATCAGGCAGGCCGTGAAAC	GGTACCGATCGTCGTCTTCGCTTTTGG	Over-expression
CC15640F1	LOC_Os01g15640	GTGTGTATATACAAGCATGAACCA	AAACTGGTTCATGCTTGTATATAC	CRISPR-CAS9
CC15640F2	LOC_Os01g15640	GTGTCAGGCTGTTCGGATGCTCGA	AAACTCGAGCATCCGAACAGCCTG	CRISPR-CAS9
1564-deltaN F	LOC_Os01g15640	GGGAGATCTGATACGGAGTACTTGTTGCC	CGTCGACTAATTATCAGCAGCTTCATTCGA	Yeast self-activation assay
1564full-R	LOC_Os01g15640	GTTCCCGGGCATGGAGTCTTTACGTGACATG	CGTCGACTAGGTATGCTGCTGATCTCTC	Yeast self-activation assay
1564R7	LOC_Os01g15640	GTTCCCGGGCATGGAGTCTTTACGTGACATG	CGTCGACTATCGAGCATCCGAACAGCCTG	Yeast self-activation assay
1564R4	LOC_Os01g15640	GTTCCCGGGCATGGAGTCTTTACGTGACATG	CGTCGACTAATTATCAGCAGCTTCATTCGA	Yeast self-activation assay
CC1564N2Y2	LOC_Os01g15640	GGCAGATCTCGATGGAGTCTTTACGTGACATGG	GCCCTGCAGCTAGCTGGCGAGAACAAAAGTC	Yeast self-activation assay
pETMalc-H-NTL3deltaC	LOC_Os01g15640	GCCTCGAGATGGAGTCTTTACGTGACATG	GCCTCGAGCTACTGAATTCCACTCCAAACATCG	Protein Purification
bZIP74-UB	LOC_Os06g41770	GCCGTACTCACGGAAACCCT	CTAGCAAGCAGCTGCTGCTAAA	qRT-PCR
bZIP74-SB	LOC_Os06g41770	CCATGCAGGAGTCTGCCGCTG	CTAGCAAGCAGCTGCTGCTAAA	qRT-PCR
OsCRT	LOC_Os03g61670	AGAGGCTTGAGGAGGAATCTGC	CTGATTCAGTTGCCTCTTCGTC	qRT-PCR
OsCRT3A	LOC_Os01g67054	GTGACGATCCGGAATATGCAAG	CCAGCTTCTCCTAGGAAGAAAG	qRT-PCR
OsPDIL2-3	LOC_Os09g27830	GAGAAGCAAGTTGGAGTTGGTG	CCGCCCAAATTGTACAGTTGAC	qRT-PCR
OsBIP1	LOC_Os02g02410	AAGAAGGTGAAGGAGCGGAT	ATTGGCTGTCTCCTTTGAATC	qRT-PCR
OsHSP	LOC_Os02g52150	TGCTCAAGGTGGTCGTGCCC	CCAAAACTTTTATCTCCATAACTT	qRT-PCR
NAC protein	LOC_Os05g34830	TGCAGAGCCAGCCCAAGATC	GCTTGCCCCAGTACATGAGG	qRT-PCR
zinc finger protein ZAT8	LOC_Os01g62190	TCGGAGCCTGAGTTACTTAA	CATCACGCTCCAAAATTTAG	qRT-PCR
RAP2-12	LOC_Os03g08460	GCAGCAGCCTATGCAGATCAA	ACAGGGGCACCATGTTTCTG	qRT-PCR
ZAT12	LOC_Os05g02390	TCGACCTGAACCATCCACCCT	ATCCGGCAAAGAGAGCTAGT	qRT-PCR
calmodulin-like protein 4	LOC_Os03g53200	GTTTTATCTCCCCAACTGAG	ATTCATCGTAGTTCACCTGCC	qRT-PCR
EIN3	LOC_Os07g48630	ATTTCTTCATCCGCGACGAC	AGTTGGATCCGGCGTACTTC	qRT-PCR
Os06g0622700	LOC_Os06g41770	CGACGAGTTCTTGCTTTCGG	CTCTTCGACGGACGTTAGCC	ChIP-qPCR
Os01g0839100	LOC_Os01g62190	CTCTGTTGGAGCCAGTCGAA	GCGCACACATGGTTTATCGG	ChIP-qPCR
Os03g0182800	LOC_Os03g08460	ACCTGCAACCAACTTTTGCC	ATGTGGGGATGGGCTGTCTA	ChIP-qPCR
Os07g0685700	LOC_Os07g48630	CGGAGTGTACGCAGCAGTAT	GTGGTATCTCTCCTCGTGCG	ChIP-qPCR
Os01g0328400	LOC_Os01g22490	ACCACTTTCGAACCGCCACTACT	ACGCCTAAGCCTGCCTGGTT	ChIP-qPCR
Os12g0113700	LOC_Os12g02210	TTCTGACACGTGACCGTCCT	CTGTGAGATGCTCTGCTTCCA	ChIP-qPCR
pNTL3-A	LOC_Os01g15640	GCGTCGACCATGATATAGCACGATGTCT	CGGGATCCTTGGGTCGTTTGCTTAACGC	Dual luciferase assay
pNTL3-B	LOC_Os01g15640	GCGTCGACCATGATATAGCACGATGTCT	CGGGATCCGGTTGTTATGTGAAACGTACC	Dual luciferase assay
pNTL3-C	LOC_Os01g15640	GCGTCGACACATTCACTTGTTAACACGC	CGGGATCCTTGGGTCGTTTGCTTAACGC	Dual luciferase assay
pNTL3-D	LOC_Os01g15640	GCGTCGACCATGATATAGCACGATGTCT	CGGGATCCGTAGACTCAAATCGCTTTCGG	Dual luciferase assay
pbZIP74-FULL	LOC_Os06g41770	GCGTCGACCTCTGGCAACACCGCTATCT	CGGGATCCCGCGATGGAGATATTTTTGT	Dual luciferase assay
pbZIP74-1	LOC_Os06g41770	GCGTCGACGTATCGGAGTATTAGCCAAT	CGGGATCCCGCGATGGAGATATTTTTGT	Dual luciferase assay
pbZIP74-2	LOC_Os06g41770	GCGTCGACGGCAACCGATCTATCCCAAT	CGGGATCCCGCGATGGAGATATTTTTGT	Dual luciferase assay
pbZIP74-3	LOC_Os06g41770	GCGTCGACCATCCTGGTCTTGCAAAACC	CGGGATCCCGCGATGGAGATATTTTTGT	Dual luciferase assay
pbZIP74-4	LOC_Os06g41770	GCGTCGACCTCTGGCAACACCGCTATCT	CGGGATCCCCGAGACAGTATAAATCAC	Dual luciferase assay
pbZIP74-5	LOC_Os06g41770	GCGTCGACGGCAACCGATCTATCCCAAT	CGGGATCCGCCTTGGGACTCCATTTGAG	Dual luciferase assay
OsbZIP17△C	LOC_Os05g34050	GCGTCGACATGGCGGAGCCGGCCCTGCT	GCGGTACCTCACTTCTTGGTCTTGGTCTTGC	Dual luciferase assay
OsbZIP16△C	LOC_Os07g44950	GCGTCGACATGGCGGAGCCGGACCTACT	GCGGTACCCTATGCAACCTTCTTGGTCTTCC	Dual luciferase assay
OsbZIP74A	LOC_Os06g41770	CGGAATTCATGGATGTAGAGTTCTTCGCC	CGGGATCCCTAGCAAGCAGCTGCTGCTAAA	Dual luciferase assay
OsNTL3△C	LOC_Os01g15640	CCCCCGGGATGGAGTCTTTACGTGACA	GCTCTAGACTACTGAATTCCACTCCAAACATCG	Dual luciferase assay
NTL3-GFP	LOC_Os01g15640	CCCCCGGGATGGAGTCTTTACGTGACA	TTTAGATCTAGAGGCTAGGTATGCTGCTGATC	Subcellular localization
NTL3-deltaC	LOC_Os01g15640	TTCAGATCTATGGAGTCTTTACGTGACATGG	GCTCTAGACTACTGAATTCCACTCCAAACATCG	Subcellular localization
CC1564-F10	LOC_Os01g15640	CGAATGAAGCTGCTGATAATGG	NA	Sequencing
1564-fl-R9	LOC_Os01g15640	NA	AGATCAGCAGCATACCTAGC	Sequencing
1564-fl-F9	LOC_Os01g15640	CCATGGAGTCTTTACGTGACA	NA	Sequencing
CC1564-R10	LOC_Os01g15640	NA	GTGCGATGTTCCAACTCAGG	Sequencing
ZIP74Fmcx	LOC_Os06g41770	CCGAGCATCTCCGACGAGT	NA	Sequencing
Z74-cxR	LOC_Os06g41770	NA	AGCCACCTCATTCTCCTTCCCGG	Sequencing
ZIP17Fmcx	LOC_Os05g34050	CAACGACTCCGTCGTCTCG	NA	Sequencing
ZIP17Rmcx	LOC_Os05g34050	NA	TGCATACCCAGGCATCCAT	Sequencing
ZIP16Fmcx	LOC_Os07g44950	GTCCGTGATCCCATCCACC	NA	Sequencing
ZIP16Rmcx	LOC_Os07g44950	NA	CAATCCAAGCAGGCTAACACC	Sequencing
